# Analysis of CFTR mutation spectrum in Yugra region (Russian Federation)

**DOI:** 10.18699/vjgb-26-78

**Published:** 2026-09

**Authors:** M.Yu. Donnikov, A.V. Morozkina, L.V. Kovalenko, N.A. Donnikova, V.V. Mescheryakov

**Affiliations:** Medical Institute of Surgut State University, Surgut, KHMAO-Yugra, Russia; Medical Institute of Surgut State University, Surgut, KHMAO-Yugra, Russia; Medical Institute of Surgut State University, Surgut, KHMAO-Yugra, Russia; Surgut Regional Clinical Center for Protection of Maternity and Childhood, Surgut, KHMAO-Yugra, Russia; Medical Institute of Surgut State University, Surgut, KHMAO-Yugra, Russia

**Keywords:** cystic fibrosis, CFTR gene, common and rare mutations, KHMAO-Yugra, муковисцидоз, ген CFTR, частые и редкие мутации, ХМАО-Югра

## Abstract

Rapid progress in both molecular diagnostics tools and targeted therapy for cystic fibrosis (CF) requires clear understanding of CFTR mutation spectrum in population constantly living in any large region of any country. There are still no published results of comprehensive genetic study of CF patients from the Yugra region (Western Siberian part of Russia). Three-step molecular genetic approach was used for gDNA samples derived from a group of 54 CF patients living in the Yugra region: (1) AFLP/RFLP was used for the panel of 35 major CFTR mutations; (2) NGS, for full sequencing of exons and exon-intron boundaries of CFTR gene; and (3) the MLPA technique, to search for large gene rearrangements. The search of major CFTR mutations in multiethnic Yugra population accounted for only 76.6 % of all CF-causing mutations. Most common were variants F508del (rs113993960), 1677delTA (rs121908776), CFTRdele2,3, E92K (rs121908751), R1066C (rs78194216). Genomic DNA samples from 19 CF patients from the tested group in which only one mutant allele was identified were analyzed by the NGS approach followed by MLPA. This allowed to reveal 19 rare pathogenic CFTR variants, including five new variants absent in CF mutation databases. Generally, three-tier molecular genetics approach applied for the first time for a Yugra-originated cohort of CF patients allowed to find 12 missense mutations, 9 nonsense ones, 6 in/dels, 4 splice-site variants, 1 CNV. Three alleles remained unidentified. Deciphering the genetic background for CF patients living in Yugra allowed to support rational administration of CFTR modulators with profound clinical effect. The need to develop a region-specific testing panel for common CFTR mutations is demonstrated.

## Abstract (ru)

Быстрый прогресс как в молекулярной диагностике, так и в таргетной терапии муковисцидоза (МВ) требует четкого понимания спектра мутаций в гене CFTR у населения, постоянно проживающего в крупном регионе любой страны. До сих пор нет опубликованных результатов ни одного комплексного генетического исследования пациентов с МВ из ХМАО-Югры (Западно-Сибирская часть России). Образцы геномной ДНК, полученные от группы из 54 пациентов с МВ, проживающих в Югре, были изучены с использованием трехэтапного молекулярно-генетического подхода: 1) ПДРФ-анализ для панели из 35 мажорных мутаций в гене CFTR; 2) NGS для полного секвенирования экзонов и экзон-интронных границ гена CFTR; 3) метод MLPA для поиска крупных генных перестроек. Поиск мажорных мутаций в гене CFTR в многоэтнической популяции Югры позволяет выявить лишь 76.6 % от всех мутаций, вызывающих МВ. Наиболее распространенными являются варианты F508del (rs113993960), 1677delTA (rs121908776), CFTRdele2,3, E92K (rs121908751), R1066C (rs78194216). Геномная ДНК 19 пациентов с МВ из исследуемой группы, у которых идентифицирован только один мутантный аллель, была проанализирована с помощью метода NGS с последующим исследованием методом MLPA. В результате выявлены 19 редких патогенных вариантов гена CFTR, включая пять новых вариантов, информация о которых отсутствует в базах данных мутаций гена CFTR. В целом трехступенчатый молекулярно-генетический подход, впервые примененный для когорты пациентов с МВ из ХМАО-Югры, позволил обнаружить 12 миссенс- мутаций, 9 нонсенс-мутаций, 6 инсерций/делеций, 4 варианта сайта сплайсинга и 1 CNV. Три аллеля остались неидентифицированными. Расшифровка генетического груза пациентов с МВ, проживающих в ХМАО-Югре, позволяет обосновать рациональное назначение модуляторов белка CFTR с выраженным клиническим эффектом. Показана необходимость разработки регион-специфической панели тестирования на распространенные мутации в гене CFTR.

## Introduction

Yugra is officially known as Khanty-Mansi Autonomous Okrug – Yugra and is located in the Western Siberian part of the Russian Federation, with a predominantly urban population of 1.7 million people scattered over an area of 534,000 km2 in several large towns and a string of small settlements. The indigenous population (Khanty and Mansi) currently comprises only 27,000 people. Rapid development of oil and gas production over the last 40 years led to radical changes in population structure. The incoming migrant population is characterized by ethnic heterogeneity and a high proportion of Turk-originated and Finno-Ugric cohorts with a predominance of people of Slavic origin. The formation of an ethnically and genetically heterogenous population creates a need of genetic studies aimed to reveal regional features of hereditary disorders.

Among these, cystic fibrosis (CF, OMIM #219700) is one of the most frequent genetic conditions with an autosomal recessive type of inheritance; it is common among Europeans and residents of North America (Ong, Ramsey, 2023; Amaral, Pankonien, 2025). CF is a multiple organ pathology caused by mutations in the CFTR gene encoding the chloride channel (Callebaut et al., 2018). Until recently, the diagnosis of CF was determined by characteristic clinical manifestations of the respiratory system and exocrine glands, indicating the onset of irreversible pathological changes (Sosnay et al., 2017b). With the spread of neonatal (newborn) screening (NBS), the diagnosis of CF becomes presymptomatic (Farrell et al., 2017), which largely determines the early initiation of therapy, prevents the development of severe complications, and improves prognosis.

Currently, more than 2,300 mutations of the CFTR gene are known (Stepanova et al., 2025), and their spectrum is strongly population-specific, which makes it difficult to correctly diagnose CF in heterogeneous populations (Petrova et al., 2020; Ayupova et al., 2024). A complete genetic diagnosis is of particular relevance in connection with optimized genetic counseling and facilitation of mutation-specific targeted CF therapy in clinical practice (Parisi et al., 2025; Terlizzi, Lopes- Pacheco, 2025). The goal of CF genetic diagnostics is to detect at least one clinically significant mutation in the CFTR gene in the majority of patients with clinical symptoms of CF (Sosnay et al., 2017a), which is ensured by using a panel of frequent (major) mutations. Federal genetic centers in the Russian Federation use panels of 33 mutations characteristic of the Russian population (Petrova et al., 2020), which identify up to 85 % of mutant CF alleles as described previously (Petrova et al., 2016). The search for the second mutation for patients from remote Yugra locations was until recently hampered by a number of resource and logistics issues and thus was performed mostly retrospectively.

According to annually updated data from CF regional registry, there are 54 patients with confirmed CF diagnosis (as of June 2025), and each year this number is gradually increased by 3–4 children. Since 2006, CF has been one of the five diseases monitored through newborn screening (NBS) program in Russia, and CF diagnostics begins immediately after birth by continuous screening examination (Therrell et al., 2024). In Yugra, as throughout Russia, mass screening of newborns for CF has been carried out as part of a national project, an important component of which is the genetic diagnostics of CF. The results of many years of continuous research make it possible to calculate and predict the incidence of CF, assess the effectiveness of the genetic diagnostics of CF NBS. In this regard, there is a strong need to study the structure of CFTR mutations in a cohort of patients in a given territory, which determines the necessity for a complete examination of patients using a method of gene diagnostics available for regional conditions. Thus, the aim of the study was to describe the range of CFTR mutations revealed in a local Russian region characterized by a small general population scattered over a large territory and having a healthcare system capable of applying modern genetic technologies to study hereditary diseases like CF.

## Materials and methods

The study was approved by the Local Ethics Committee of the Medical Institute of Surgut State University (protocol No. 6, February 12, 2025). All participants (or their official representatives) gave their informed consent. Clinical and demographic characteristics of the studied CF patients living in Yugra are listed in Table 1. All EDTA blood samples were retrieved as frozen aliquots from the regional biobank located in the Medical Institute of Surgut State University.

**Table 1.**
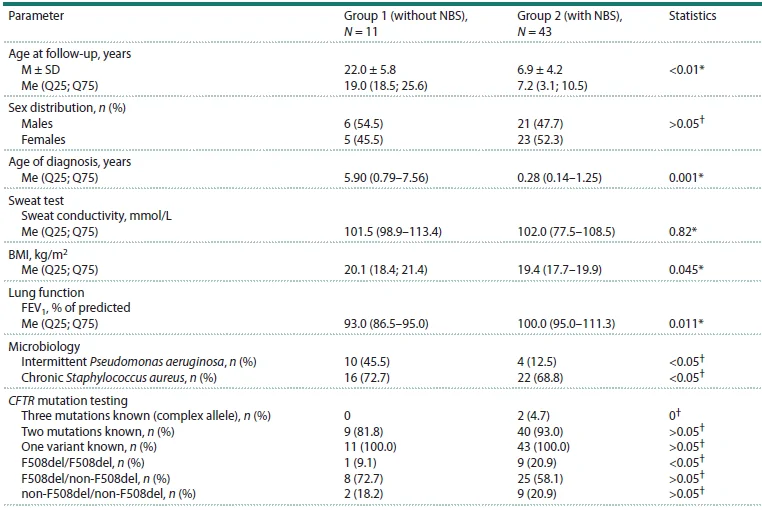
Demographic, clinical and brief genetics characteristic of the CF patients studied * Mann–Whitney U test (when comparing median values). † Fischer’s exact test (when comparing %).

AFLP/RFLP. In-house amplified fragment length (AFLP) and restriction fragment length polymorphism (RFLP) techniques were used to detect insertion/deletion variants and nucleotide substitutions, respectively (Ivaschenko et al., 1995). The panel included 35 CF-causing mutations (legacy names given) relevant for the North-Western region of Russia: F508del (rs113993960), I507del (rs121908745), CFTRdele2,3, E92K (rs121908751), 3849+10kb C>T (rs75039782), 1677delTA (rs121908776), N1303K (rs80034486), W1282X (rs77010898), L138ins (rs397508686), G542X (rs113993959), 394delTT (rs121908769), R334W (rs121909011), W1282R (rs766509028), S1196X (rs886061212), 2143delT (rs121908812), 1367del5 (rs397508184), 2183AA>G (rs121908799), R1066C (rs78194216), W1310X (rs397508645), 712-1G>T (rs121908793), 621+1G>T (rs78756941), R553X (rs74597325), L1335P (rs397508658), 4015delA (rs1553429051), R1162X (rs74767530), 3120+1G>A (rs75096551), 2184insA (rs121908769), 2184delA (rs121908798), 2113delA, 2118del4, 2176insC, 2183delAA (rs121908769), R347P (rs77932196), G551D (rs75527207), W1282G. In cases where only one CFTR mutation was found, we performed NGS of the entire gene. Positive findings were confirmed in parents to determine mutant allele segregation.

Next generation sequencing (NGS) was performed on the MiSeq platform (Illumina, USA) with library preparation based on NimbleGen SeqCap EZ Choice: 151012_HG38_ CysFib_EZ_HX3, fully covering the CFTR gene. Reference genome NCBI37/hg19 was used for NGS data analysis. Genetic variants were identified by BWA (http://bio-bwa.source forge.net/), GATK (https://www.broadinstitute.org/gatk/), SNPeff (http://snpeff.sourceforge.net/), Basespace Illumina (https://basespace.illumina.com/apps/). Variant pathogenicity was assessed using data from Exome Aggregation Consortium (http://exac.broadinstitute.org/), Exome Variant Server (http:// evs.gs.washington.edu/EVS/), 1000 Genomes Project (http:// browser.1000genomes.org/index.html), dbSNP (http://www. ncbi.nlm.nih.gov/SNP/), ClinVar (http://www.ncbi.nlm.nih. gov/clinvar/), OMIM (http://omim.org/), HGMD (http://www. hgmd.cf.ac.uk/ac/index.php). Final results were verified by direct Sanger sequencing using an ABI 3130 genetic analyzer (Applied Biosystems, USA).

MLPA. A search for large rearrangements (deletions/duplications – CNV) involving the CFTR locus in chromosome region 7q31.2 was performed using multiplex ligation-dependent probe amplification (MLPA) in cases where no pathogenic variant was found. MLPA was performed using SALSA MLPA probemix P091-D2 CFTR (MRC-Holland, the Netherlands) according to the manufacturer’s recommendations. The MLPA results were analyzed using Coffalyser.Net (MRC-Holland).

To assess the disease course in patients living in Yugra and included in regional CF registry, the following key clinical parameters were taken into account: the patient age at followup (first half of 2025), sweat chloride concentration (mmol/L), body mass index (BMI) (kg/m2), lung function estimation (FEV1, % predicted), microbiological status of the lower respiratory tract.

Statistical analysis was performed using the STATISTICA 8.0 program. To compare the observed categorical variables, the Fisher test was used, while for quantitative tests, the Mann–Whitney test was utilized. Results were considered significant at p ≤ 0.05.

## Results

As of June 2025, 54 CF patients were officially registered in Yugra (Table 1). The long-term mean annual prevalence (2016–2024) was 48.6 cases, and the incidence was one case per 9,025 newborns per year, which corresponds to nationwide data (1:9,000) (Registry…, 2025).

The diagnosis of CF was confirmed according to the guidelines of the European Cystic Fibrosis Society as well as the Russian National Consensus on Cystic Fibrosis: for adult patients, the presence of typical pulmonary or gastrointestinal symptoms was established, for patients with NBS, at least one of the following was found: two positive sweat chloride tests, or the identification of two CFTR mutations in trans (Kondratyeva et al., 2019; Castellani et al., 2023).

The studied cohort consisted of 54 patients, mostly children (45 individuals) and 9 adults (the oldest was born in 1988). To demonstrate NBS efficiency, we subdivided the cohort in two subgroups depending on NBS availability (Table 1). All of the known CF patients residing in Yugra and included in regional CF registry were genetically studied in consecutive steps. As the first step, we used the testing panel of our colleagues from St. Petersburg (Glotov O., personal communication) developed for routine testing of CF-suspected individuals living in Northwestern Russia. Samples with unidentified mutant alleles underwent NGS of the CFTR gene. As the last step, MLPA was performed to confirm or identify large gene rearrangements.

Analysis of the patient cohort using a panel of 35 CFTR mutations common in Northwestern Russia revealed a particularly high frequency of two mutations in the Yugra population: c.1521_1523delCTT (p.Phe508del; F508del) – 53/111 alleles (47.7 %), and c.1545_1546delTA (p.Tyr515X; 1677delTA) – 8/111 alleles (7.2 %) (Table 2). Overall, only 15 out of the 35 mutations included in the panel were detected (Table 2), whereas 20 mutations were not found at all. Thus, the overall efficiency of the panel was detection of 85/111 alleles (76.6 %), which is less than that for a similar panel developed by our colleagues from Moscow (83 %); it obviously needs further customization for regional-specific CFTR variants (Petrova et al., 2020).

**Table 2.**
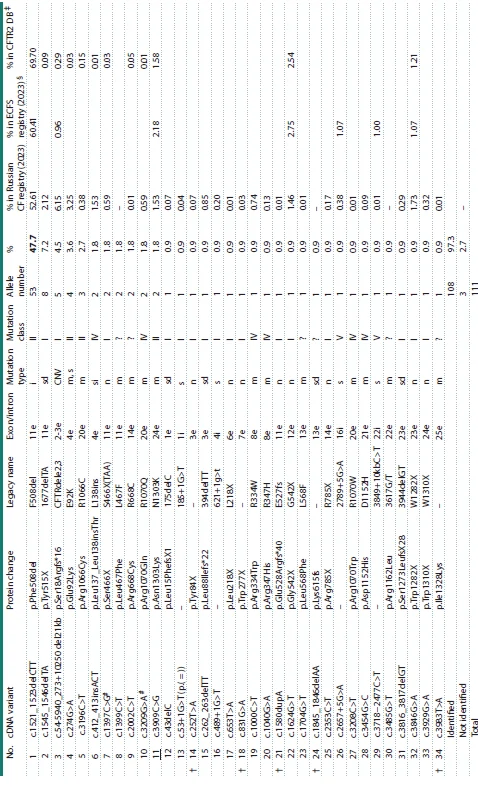
The CFTR gene variants identified in Yugra-living patients Note. Mutation types used: (m) – missense mutation, (n) – nonsense mutation, (sd) – frameshift deletion, (si) – frameshift insertion, (s) – splice-site, (i) – in-frame insertion, (CNV) – copy number variation (large rearrangement). # Complex allele S466X-R1070Q. † Newly identified CFTR variants. § Out of 7 most frequent (Zolin et al., 2025). ‡ Out of 6 most frequent.

Sequencing of all the samples confirmed the results of panel testing and also additionally identified 19 rare mutations, 15 of which were unique (reported in individual families). Using the MLPA method, only one type of large rearrangement of the CFTR gene (c.54-5940_273+10250del21kb, CFTRdele2,3) was found in 5 unrelated patients, which constitutes 5/111 (4.5 %) of all tested mutant alleles. In three patients (two adults and one kid) with a confirmed CF diagnosis, only one mutation was revealed despite the use of NGS and MLPA. Thus, three mutant alleles remain unidentified and require further in-depth study.

As a result, 34 pathogenic genetic variants in the CFTR gene were detected (Table 2). Among these, there are 12 missense mutations, 9 nonsense mutations, 5 frameshift deletions, 1 frameshift insertion, 4 splice-site mutations, 1 in-frame insertion, and 1 CNV. Frequent mutation E92K (prevalent for Chuvash nationality) was attributed to both missense and splice-site mutation classes.

Mutation F508del (c.1521_1523delCTT) is among the most common CFTR variants worldwide (Bobadilla et al., 2002), and it is the most frequent in Russian CF patients (52.61 % in the 2023 registry (Registry…, 2025)). Its frequency in Yugra (47.7 %) precedes the lowest number observed in the North Caucasian region of Russia (27.1 %). F508del homozygotes were observed in 10 patients, F508del in a heterozygous compound state was observed in 33 patients (61.1 %).

The second most frequent mutation identified (1677delTA) was described previously as common for Georgian patients (Petrova et al., 2021); but in Yugra, these eight alleles are distributed among patients of Chechen origin (two siblings and one unrelated proband are homozygous and two unrelated patients are heterozygous for this variant). These cases changed the expected distribution of CFTR mutations within the Yugra population, in spite of the fact that the Chechens comprise only 0.41 % of the Yugra population structure (Rosstat data, available online). A possible explanation is health-related internal migration due to a high level of medical care for CF patients in Yugra compared to North Caucasus. Before these families relocated to Yugra, the second most frequent variant had been the Slavic-associated CFTRdele2,3 variant.

The reported predominant distribution of the CFTRdele2,3 (c.54-5940_273+10250del21kb) variant for Eastern European populations (Dörk et al., 2000) is confirmed in our study, but compared to the All-Russian CF registry, this mutation is the third by frequency in Yugra: 5/111 alleles (4.5 % vs 6.15 %, respectively), preceded by the 1677delTA mutation.

The fourth most frequent mutation is E92K (c.274G>A) – 4/111 alleles (3.6 %). It has been described in the Chuvash population (Stepanova et al., 2012). In our study all four alleles were detected in the heterozygous state in unrelated patients of Russian origin.

The fifth most frequent mutation is R1066C (c.3196C>T) – 3/111 alleles (2.7 %) – revealed in a homozygous state in one CF patient and in one unrelated heterozygous patient, both of non-Slavonic nationalities (Azerbaijani and Dagestan).

Five other relatively frequent mutations (L138ins, S466X(TAA), R1070Q, L467F, R668C) were observed in 2/111 alleles (1.8 %) each in a compound heterozygous state. Moreover, S466X(TAA) mutation formed a complex allele with R1070Q, which was revealed in two siblings. L138ins was also revealed in two siblings. N1303K was found in a homozygous state in one proband. The remaining 23 mutant alleles were private and were observed only once in a compound heterozygous state.

Additionally, five novel variants absent from the main CF mutation databases (CFTR1, CFTR2, ExAC) were identified for the first time: three of them are nonsense mutations (c.252T>A (p.Tyr84X), c.831G>A (p.Trp277X), c.1580dupA (p.Glu528Argfs*40)), one mutation is missense (c.3983T>A (p.Ile1328Lys), and one mutation is a frameshift deletion (c.1845_1846delAA). Clinical significance of these newly found mutations is estimated as “likely pathogenic” (Zhang et al., 2020) and requires additional functional study. All five children carrying these variants in trans with F508del had high sweat chloride values and a pancreatic-insufficient phenotype with pulmonary manifestations typical of CF.

Over half of the studied alleles (62) corresponding to a relatively low variety of mutations per se (only four – F508del, E92K, R1066C, N1303K) are attributed to the II class of CFTR mutation. Among these, ten patients were homozygous and 33 patients were heterozygous for F508del. That makes a significant number of patients (at least 44) carrying these alleles eligible for targeted drug therapy. Currently, 36 children with CF living in Yugra are receiving targeted therapy and this number is rapidly growing. Unfortunately, there are 28 mutant alleles belonging to the I class (and carried by 10 patients) with potential targeted drugs still in development.

## Discussion

According to the Russian Registry of CF patients in 2023 (RF CF Registry), among at least 275 pathogenic CFTR variants, only eleven mutations are the most frequent ones in Russia with frequencies exceeding 1 % in the tested sample: F508del shares 51.4 %, CFTRdele2,3 – 6.1 %, E92K – 3.7 %, 1677delTA – 2.5 %, 3849+10kbC>T – 2.2 %, 2143delT – 2.0 %, 2184insA – 1.9 %, W1282X – 1.7 %, L138ins and N1303K each sharing 1.7 %, G542X – 1.5 % (Registry…, 2025). It is conceivable that the spectrum of CFTR mutations varies in different regions depending on specific ethnic background of the population. The East Slavic population comprises 77.7 % of the population living in Russia, according to the 2010 census. Moreover, in the European part, Russians comprise 85–90 % of the population (Rosstat data). In Yugra, top three nationalities according to the latest statistical Rosstat data are: Slavonic (Russians, Ukrainians and Belarusians) – 74.1 %, Tatar – 6.3 %, Bashkir – 2.3 %. Indigenous people (Khanty and Mansi) comprise a small part: 1.5 and 0.9 %, respectively. To our knowledge, no CF patients of indigenous origin have been identified to date, in spite of equal involvement of all newborn children in neonatal screening.

A comparison of frequency profiles for 34 CFTR mutations revealed in Yugra with that of reported officially by Russian CF registry (data for 2023 (Registry…, 2025)) reveals a similarity of frequency distributions for Yugra patients and for patients from the All-Russian sample (Table 2), which is not surprising as ethnic Russians make up the majority of CF patients in the Russian Federation. The differences between values are determined by the much larger sample size in Russian CF registry than in Yugra regional CF registry (4,058 and 54 patients, respectively). Any small change in patient number reported in Yugra (for example, during updating genetics data for newly found newborns with CF) will inevitably cause the range of frequent regional mutations to be restructured. Comparison of mutation profiles in Yugra with those from European CF registry (Zolin et al., 2025) and the CFTR2 database (Sosnay et al., 2017a) appears to be inappropriate (see Table 2), except for the F508del mutation. This may reflect greater differences in population structures between Western Europe and Eastern territories of Russia.

## Conclusion

The relatively small (1.78 million) size of the population constantly living in the Yugra region and its highly developed healthcare system allowed us to perform comprehensive genetic testing for all 54 CF patients currently included in regional CF registry. The shaping of CF patients’ cohort in Yugra actually is the result of efficient NBS efforts which allowed to sieve up to 396,000 kids born since 2006 in terms of biochemical analysis of immunoreactive trypsinogen levels and sweat testing. Also, a comprehensive genetic analysis of CF patients became possible with the advent of NGS in routine laboratory work. Targeted CF therapy with CFTR modulators that started in 2022 in Yugra greatly improved both quality of life and prognosis for patients. The three-step model of genetic testing proposed by leading Russian geneticists (Petrova et al., 2020) and the results of the current study, which demonstrated a relatively low efficiency of using a panel of CFTR mutations not adapted to a regional population structure, clearly indicate the need for development of a region-focused panel for the preliminary search for most frequent mutations in Yugra-born patients as the 1st-tier genetic test. Comprehensive genetic study of CFTR mutations shows that out of 34 identified pathogenic variants, only 11 mutant alleles had the frequencies exceeding 1 %, whereas the remaining 23 variants were unique and were observed only in individual cases. This fact strongly supports the usefulness of consistent use of NGS and MLPA for identifying rare CFTR mutations. Functional analysis for novel genetic variants is also recommended.

The obtained genetic information is useful for further improvement of medical care in CF patients and related families with regard to effective genetic counseling and resource allocation in cases where CF-targeted drug therapy with CFTR modulators is available. Identification of unknown pathogenic variants contributes to the general knowledge about CFTR mutations heterogeneity at the national and worldwide scale.

## Conflict of interest

The authors declare no conflict of interest.

## References

Amaral M.D., Pankonien I. Cystic fibrosis as a paradigmatic disease
in bringing science to the bedside. FEBS Lett. 2025;599(22):3196-
3207. doi 10.1002/1873-3468.70101

Ayupova G., Litvinov S., Akhmetova V., Minniakhmetov I., Mokrysheva
N., Khusainova R. Population characteristics of the spectrum
and frequencies of CFTR gene mutations in patients with cystic fibrosis
from the Republic of Bashkortostan (Russia). Genes (Basel).
2024;15(10):1335. doi 10.3390/genes15101335

Bobadilla J.L., Macek M., Jr., Fine J.P., Farrell P.M. Cystic fibrosis:
a worldwide analysis of CFTR mutations – correlation with incidence
data and application to screening. Hum Mutat. 2002;19(6):575-606.
doi 10.1002/humu.10041

Callebaut I., Chong P.A., Forman-Kay J.D. CFTR structure. J Cyst
Fibros.
2018;17(2S):S5-S8. doi 10.1016/j.jcf.2017.08.008

Castellani C., Simmonds N.J., Barben J., Addy C., Bevan A., Burgel
P.R., Drevinek P., … Nährlich L., Raraigh K., Sermet-Gaudelus
I., Sommerburg O., Southern K.W. Standards for the care of people
with cystic fibrosis (CF): a timely and accurate diagnosis. J Cyst
Fibros. 2023;22(6):963-968. doi 10.1016/j.jcf.2023.09.008

Dörk T., Macek M., Mekus F., Tümmler B., Tzountzouris J., Casals T.,
Krebsová A., … Stuhrmann M., Bauer I., Seydewitz H.H., Neumann
T., Jakubiczka S. Characterization of a novel 21-kb deletion,
CFTRdele2,3(21 kb), in the CFTR gene: a cystic fibrosis mutation
of Slavic origin common in Central and East Europe. Hum Genet.
2000;106(3):259-268. doi 10.1007/s004390000246

Farrell P.M., White T.B., Howenstine M.S., Munck A., Parad R.B.,
Rosenfeld M., Sommerburg O., … Wilschanski M., Sermet-Gaudelus
I., Blau H., Gartner S., McColley S.A. Diagnosis of cystic fibrosis
in screened populations. J Pediatr. 2017;181S:S33-S44.e2. doi
10.1016/j.jpeds.2016.09.065

Ivaschenko T., Bakay M., Baranov V. Novel frameshift mutation in
exon 4 of CFTR gene. Hum Mutat. 1995;5(2):184-185. doi 10.1002/
humu.1380050215

Kondratyeva E.I., Kashirskaya N.Y., Kapranov N.I. (Eds) National
Consensus Project. Cystic Fibrosis: Definition, Diagnostic Criteria,
Treatment. Moscow, 2019 (in Russian)

Ong T., Ramsey B.W. Cystic fibrosis: a review. JAMA. 2023;329(21):
1859-1871. doi 10.1001/jama.2023.8120

Parisi G.F., Terlizzi V., Manti S., Papale M., Pecora G., Presti S., Tosto
M., Leonardi S. Cutting-edge advances in cystic fibrosis: from
gene therapy to personalized medicine and holistic management.
Genes (Basel). 2025;16(4):402. doi 10.3390/genes16040402

Petrova N.V., Kondratyeva E.I., Krasovsky S.A., Polyakov A.V.,
Ivachshenko T.E., Pavlov A.E., Zinchenko R.A., … Semykin S.Y.,
Sergienko D.F., Simonova O.I., Shabalova L.A., Kashirskaya N.Y.
National Consensus Project “Cystic fibrosis: definition, diagnostic
criteria, treatment” Section “Genetics of Cystic Fibrosis. Molecular
genetic diagnosis of cystic fibrosis”. Med Genet. 2016;15(11):29-45
(in Russian)

Petrova N.V., Kashirskaya N.Y., Vasilyeva T.A., Kondratyeva E.I.,
Zhekaite E.K., Voronkova A.Y., Sherman V.D., Galkina V.A., Ginter
E.K., Kutsev S.I., Marakhonov A.V., Zinchenko R.A. Analysis of
CFTR mutation spectrum in ethnic Russian cystic fibrosis patients.
Genes (Basel). 2020;11(5):554. doi 10.3390/genes11050554

Petrova N., Balinova N., Marakhonov A., Vasilyeva T., Kashirskaya N.,
Galkina V., Ginter E., Kutsev S., Zinchenko R. Ethnic differences
in the frequency of CFTR gene mutations in populations of the
European
and North Caucasian part of the Russian Federation. Front
Genet. 2021;12:678374. doi 10.3389/fgene.2021.678374

Registry of Patients with Cystic Fibrosis in the Russian Federation.
2023. Moscow, 2025. doi 10.61726/1981.2025.26.16.001 (in Russian)

Sosnay P.R., Salinas D.B., White T.B., Ren C.L., Farrell P.M., Raraigh
K.S., Girodon E., Castellani C. Applying cystic fibrosis transmembrane
conductance regulator genetics and CFTR2 data to facilitate
diagnoses. J Pediatr. 2017a;181S:S27-S32.e1. doi 10.1016/
j.jpeds.2016.09.063

Sosnay P.R., White T.B., Farrell P.M., Ren C.L., Derichs N., Howenstine
M.S., Nick J.A., De Boeck K. Diagnosis of cystic fibrosis in
nonscreened populations. J Pediatr. 2017b;181S:S52-S57.e2. doi
10.1016/j.jpeds.2016.09.068

Stepanova A.A., Abrukova A.V., Savaskina E.N., Poliakov A.V.
Mutation p.E92K is the primary cause of cystic fibrosis in Chuvashes.
Russ J Genet. 2012;48:731-737. doi 10.1134/S102279541
2060166

Stepanova A.A., Galeeva N.M., Schagina O.A., Ryzhkova O.P., Krasovsky
S.A., Voronkova A.Y., Zhekaite E.K., Melyanovskaya Y.L.,
Kondratyeva E.I., Polyakov A.V. Results of the extended genotyping
program for patients with suspected cystic fibrosis. Med Genet.
2025;24(7):100-103. doi 10.25557/2073-7998.2025.07.100-103 (in
Russian)

Terlizzi V., Lopes-Pacheco M. Cystic fibrosis: new challenges and
perspectives
beyond elexacaftor/tezacaftor/ivacaftor. Ther Adv
Respir Dis. 2025;19:17534666251323194. doi 10.1177/17534666
251323194

Therrell B.L., Padilla C.D., Borrajo G.J.C., Khneisser I., Schielen
P.C.J.I., Knight-Madden J., Malherbe H.L., Kase M. Current status
of newborn bloodspot screening worldwide 2024: a comprehensive
review of recent activities (2020–2023). Int J Neonatal Screen.
2024;10(2):38. doi 10.3390/ijns10020038

Zhang J., Yao Y., He H., Shen J. Clinical interpretation of sequence
variants. Curr Protoc Hum Genet. 2020;106(1):e98. doi 10.1002/
cphg.98

Zolin A., Adamoli A., Bakkeheim E. ECFSPR Annual Report 2023.
European
Cystic Fibrosis Society, 2025. Available: https://www.ecfs.
eu/sites/default/files/Annual%20Report_2023_vs1.2_ECFSPR_
20250721.pdf

